# Correction: Long-term fish assemblages of the Ohio River: Altered trophic and life history strategies with hydrologic alterations and land use modifications

**DOI:** 10.1371/journal.pone.0218915

**Published:** 2019-06-20

**Authors:** Mark Pyron, Meryl C. Mims, Mario M. Minder, Robert C. Shields, Nicole Chodkowski, Caleb C. Artz

In the title of this article, the words “life history strategies” should not be present. The correct title is: Long-term fish assemblages of the Ohio River: Altered trophic abundances with hydrologic alterations and land use modifications. The correct citation is: Pyron M, Mims MC, Minder MM, Shields RC, Chodkowski N, Artz CC (2019) Long-term fish assemblages of the Ohio River: Altered trophic abundances with hydrologic alterations and land use modifications. PLoS ONE 14(4): e0211848. https://doi.org/10.1371/journal.pone.0211848
