# Supplementary material for: Long-term fish assemblages of the Ohio River: Altered trophic and life history strategies with hydrologic alterations and land use modifications
Source: PLoS One. 2019 Apr 24;14(4):e0211848. doi: 10.1371/journal.pone.0211848 (PMC6481763; doi:10.1371/journal.pone.0211848)
Supplement: S1 Appendix — Strategy weights are log-transformed and classified into opportunistic (Opp), periodic (Per), and equilibrium (Equ), and hard classification (Class) includes intermediate classifications. (DOCX) [file pone.0211848.s001.docx]

**S1 Appendix.** Life history classifications based on Mims & Olden [27] and Perkin et al. [4]. Strategy weights are log-transformed and classified into opportunistic (Opp), periodic (Per), and equilibrium (Equ), and hard classification (Class) includes intermediate classifications

| Species | Genus species | Class | Opp | Per | Equ |
| --- | --- | --- | --- | --- | --- |
| Skipjack Herring | *Alosa chrysochloris* | O | 0.79 | 0.51 | 0.37 |
| Alewife | *Alosa pseudoharengus* | O | 1.04 | 0.42 | 0.36 |
| Rockbass | *Ambloplites rupestris* | I | 0.46 | 0.32 | 0.53 |
| White Catfish | *Ameiurus catus* | E | 0.38 | 0.30 | 0.64 |
| Black Bullhead | *Ameiurus melas* | E | 0.38 | 0.30 | 0.63 |
| Yellow Bullhead | *Ameiurus natalis* | E | 0.38 | 0.30 | 0.63 |
| Brown Bullhead | *Ameiurus nebulosus* | E | 0.38 | 0.30 | 0.65 |
| Bowfin | *Amia calva* | E | 0.34 | 0.34 | 3.00 |
| Eastern Sand Darter | *Ammocrypta pellucida* | O | 0.85 | 0.36 | 0.39 |
| American Eel | *Anguilla rostrata* | P | 0.56 | 1.39 | 0.36 |
| Pirate Perch | *Aphredoderus sayanus* | I | 0.46 | 0.31 | 0.50 |
| Freshwater Drum | *Aplodinotus grunniens* | O | 0.66 | 0.49 | 0.39 |
| Northern Stoneroller | *Campostoma anomalum* | O | 0.83 | 0.37 | 0.40 |
| River Carpsucker | *Carpiodes carpio* | O | 0.78 | 0.43 | 0.38 |
| Quillback | *Carpiodes cyprinus* | O | 0.80 | 0.45 | 0.38 |
| Highfin Carpsucker | *Carpiodes velifer* | O | 0.88 | 0.41 | 0.37 |
| Goldfish | *Carrasius auratus* | O | 0.78 | 0.41 | 0.42 |
| White Sucker | *Catostomus commersoni* | O | 0.71 | 0.44 | 0.39 |
| Banded Sculpin | *Cottus carolinae* | I | 0.46 | 0.32 | 0.53 |
| Grass Carp | *Ctenopharyngodon idella* | P | 0.50 | 0.90 | 0.41 |
| Blue Sucker | *Cycleptus elongatus* | I | 0.52 | 0.51 | 0.43 |
| Red Shiner | *Cyprinella lutrensis* | O | 2.23 | 0.37 | 0.34 |
| Spotfin Shiner | *Cyprinella spiloptera* | O | 0.84 | 0.36 | 0.39 |
| Steelcolor Shiner | *Cyprinella whipplei* | O | 0.84 | 0.36 | 0.39 |
| Common Carp | *Cyprinus carpio* | I | 0.65 | 0.78 | 0.36 |
| Gizzard Shad | *Dorosoma cepedianum* | O | 0.91 | 0.44 | 0.36 |
| Threadfin Shad | *Dorosoma petenense* | O | 1.37 | 0.38 | 0.35 |
| Streamline Chub | *Erimystax dissimilis* | O | 1.49 | 0.38 | 0.34 |
| Gravel Chub | *Erimystax x-punctatus* | O | 1.84 | 0.37 | 0.34 |
| Northern Pike | *Esox lucius* | O | 0.61 | 0.52 | 0.40 |
| Muskellunge | *Esox masquinongy* | I | 0.46 | 0.59 | 0.45 |
| Mud Darter | *Etheostoma asprigene* | O | 0.84 | 0.36 | 0.39 |
| Greenside Darter | *Etheostoma blennioides* | O | 0.84 | 0.37 | 0.40 |
| Rainbow Darter | *Etheostoma caeruleum* | O | 0.84 | 0.36 | 0.39 |
| Bluebreast Darter | *Etheostoma camurum* | O | 0.84 | 0.36 | 0.40 |
| Fantail Darter | *Etheostoma flabellare* | I | 0.59 | 0.34 | 0.45 |
| Stripetail Darter | *Etheostoma kennicotti* | I | 0.59 | 0.34 | 0.45 |
| Johnny Darter | *Etheostoma nigrum* | I | 0.59 | 0.34 | 0.45 |
| Orangethroat Darter | *Etheostoma spectabile* | O | 0.85 | 0.36 | 0.39 |
| Tippecanoe Darter | *Etheostoma tippecanoe* | O | 0.85 | 0.36 | 0.39 |
| Variegate Darter | *Etheostoma variatum* | O | 0.84 | 0.37 | 0.40 |
| Banded Darter | *Etheostoma zonatum* | O | 0.84 | 0.36 | 0.39 |
| Banded Killifish | *Fundulus diaphanus* | O | 0.84 | 0.36 | 0.39 |
| Blackstripe Topminnow | *Fundulus notatus* | O | 0.84 | 0.36 | 0.39 |
| Western Mosquitofish | *Gambusia affinis* | I | 0.46 | 0.31 | 0.49 |
| Goldeye | *Hiodon alosoides* | O | 0.71 | 0.43 | 0.39 |
| Mooneye | *Hiodon tergisus* | O | 0.81 | 0.42 | 0.38 |
| Cypress Minnow | *Hybognathus hayi* | O | 1.77 | 0.37 | 0.34 |
| Mississippi Silvery Minnow | *Hybognathus nuchalis* | O | 1.57 | 0.38 | 0.34 |
| Bigeye Chub | *Hybopsis amblops* | O | 1.91 | 0.37 | 0.34 |
| Northern Hogsucker | *Hypentelium nigricans* | O | 0.96 | 0.40 | 0.36 |
| Bighead Carp | *Hypophthalmichthys nobilis* | I | 0.50 | 0.57 | 0.43 |
| Silver Carp | *Hypophthalmichthys molitrix* | P | 0.48 | 0.75 | 0.42 |
| Ohio Lamprey | *Ichthyomyzon bdellium* | O | 0.74 | 0.40 | 0.44 |
| Chestnut Lamprey | *Ichthyomyzon castaneus* | O | 0.81 | 0.38 | 0.41 |
| Silver Lamprey | *Ichthyomyzon unicuspis* | O | 0.74 | 0.40 | 0.44 |
| Blue Catfish | *Ictalurus furcatus* | E | 0.34 | 0.34 | 1.95 |
| Channel Catfish | *Ictalurus punctatus* | E | 0.37 | 0.31 | 0.70 |
| Smallmouth Buffalo | *Ictiobus bubalus* | O | 0.71 | 0.49 | 0.39 |
| Bigmouth Buffalo | *Ictiobus cyprinellus* | O | 0.71 | 0.51 | 0.38 |
| Black Buffalo | *Ictiobus niger* | O | 0.70 | 0.47 | 0.39 |
| Brook Silverside | *Labidesthes sicculus* | O | 1.61 | 0.37 | 0.34 |
| Spotted Gar | *Lepisosteus oculatus* | I | 0.54 | 0.48 | 0.42 |
| Longnose Gar | *Lepisosteus osseus* | I | 0.44 | 0.54 | 0.46 |
| Shortnose Gar | *Lepisosteus platostomus* | O | 0.62 | 0.46 | 0.40 |
| Green Sunfish | *Lepomis cyanellus* | I | 0.46 | 0.32 | 0.51 |
| Pumpkinseed | *Lepomis gibbosus* | I | 0.46 | 0.32 | 0.52 |
| Warmouth | *Lepomis gulosus* | I | 0.46 | 0.32 | 0.52 |
| Orangespotted Sunfish | *Lepomis humilis* | I | 0.46 | 0.32 | 0.50 |
| Bluegill | *Lepomis macrochirus* | I | 0.46 | 0.33 | 0.53 |
| Longear Sunfish | *Lepomis megalotis* | I | 0.46 | 0.32 | 0.52 |
| Redear Sunfish | *Lepomis microlophus* | I | 0.46 | 0.33 | 0.56 |
| American Brook Lamprey | *Lethenteron appendix* | O | 0.79 | 0.38 | 0.42 |
| Striped Shiner | *Luxilus chrysocephalus* | I | 0.59 | 0.35 | 0.46 |
| Common Shiner | *Luxilus cornutus* | I | 0.59 | 0.34 | 0.45 |
| Redfin Shiner | *Lythrurus umbratilis* | O | 0.85 | 0.36 | 0.39 |
| Shoal Chub | *Macrhybopsis hyostoma* | I | 0.00 | 0.37 | 0.34 |
| Silver Chub | *Macrhybopsis storeriana* | O | 1.30 | 0.38 | 0.35 |
| Mississippi Silverside | *Menidia audens* | O | 1.40 | 0.38 | 0.35 |
| Smallmouth Bass | *Micropterus dolomieu* | E | 0.45 | 0.34 | 0.64 |
| Spotted Bass | *Micropterus punctulatus* | E | 0.45 | 0.34 | 0.63 |
| Largemouth Bass | *Micropterus salmoides* | E | 0.45 | 0.34 | 0.61 |
| Spotted Sucker | *Minytrema melanops* | O | 0.74 | 0.43 | 0.38 |
| White Perch | *Morone americana* | O | 0.87 | 0.43 | 0.37 |
| White Bass | *Morone chrysops* | O | 0.80 | 0.51 | 0.37 |
| Yellow Bass | *Morone mississippiensis* | O | 0.87 | 0.41 | 0.37 |
| Striped Bass | *Morone saxatilis* | O | 0.61 | 0.30 | 0.30 |
| Silver Redhorse | *Moxostoma anisurum* | I | 0.60 | 0.44 | 0.49 |
| Smallmouth Redhorse | *Moxostoma breviceps* | O | 0.60 | 0.43 | 0.49 |
| River Redhorse | *Moxostoma carinatum* | I | 0.59 | 0.44 | 0.49 |
| Black Redhorse | *Moxostoma duquesni* | O | 0.73 | 0.40 | 0.44 |
| Golden Redhorse | *Moxostoma erythrurum* | O | 0.67 | 0.41 | 0.46 |
| Shorthead Redhorse | *Moxostoma macrolepidotum* | O | 0.62 | 0.43 | 0.48 |
| River Chub | *Nocomis micropogon* | I | 0.58 | 0.35 | 0.47 |
| Golden Shiner | *Notemigonus crysoleucas* | O | 1.76 | 0.38 | 0.34 |
| Emerald Shiner | *Notropis atherinoides* | O | 1.85 | 0.37 | 0.34 |
| River Shiner | *Notropis blennius* | O | 1.52 | 0.38 | 0.34 |
| Bigeye Shiner | *Notropis boops* | O | 2.92 | 0.37 | 0.34 |
| Silverjaw Minnow | *Notropis buccatus* | O | 1.74 | 0.37 | 0.34 |
| Ghost Shiner | *Notropis buchanani* | O | 2.08 | 0.37 | 0.34 |
| Spottail Shiner | *Notropis hudsonius* | O | 1.65 | 0.37 | 0.34 |
| Silver Shiner | *Notropis photogenis* | O | 1.73 | 0.37 | 0.34 |
| Rosyface Shiner | *Notropis rubellus* | O | 0.84 | 0.37 | 0.40 |
| Silverband Shiner | *Notropis shumardi* | O | 2.62 | 0.37 | 0.34 |
| Sand Shiner | *Notropis stramineus* | O | 2.20 | 0.37 | 0.34 |
| Mountain Madtom | *Noturus eleutherus* | I | 0.46 | 0.32 | 0.50 |
| Slender Madtom | *Noturus exilis* | I | 0.46 | 0.32 | 0.51 |
| Stonecat | *Noturus flavus* | I | 0.46 | 0.32 | 0.54 |
| Tadpole Madtom | *Noturus gyrinus* | I | 0.46 | 0.32 | 0.50 |
| Brindled Madtom | *Noturus murus* | I | 0.46 | 0.32 | 0.51 |
| Freckled Madtom | *Noturus nocturnus* | I | 0.46 | 0.32 | 0.51 |
| Northern Madtom | *Noturus stigmosus* | I | 0.46 | 0.32 | 0.51 |
| Rainbow Trout | *Onchorhynchus mykiss* | O | 0.75 | 0.39 | 0.43 |
| Pugnose Minnow | *Opsopoedus emiliae* | I | 0.59 | 0.34 | 0.45 |
| Yellow Perch | *Perca flavescens* | O | 1.06 | 0.40 | 0.36 |
| Logperch | *Percina caprodes* | O | 0.82 | 0.37 | 0.41 |
| Channel Darter | *Percina copelandi* | O | 0.85 | 0.36 | 0.39 |
| Gilt Darter | *Percina evides* | O | 0.84 | 0.37 | 0.40 |
| Blackside Darter | *Percina maculata* | O | 0.84 | 0.37 | 0.40 |
| Slenderhead Darter | *Percina phoxocephala* | O | 0.84 | 0.36 | 0.39 |
| Dusky Darter | *Percina sciera* | O | 0.84 | 0.36 | 0.39 |
| River Darter | *Percina shumardi* | O | 0.84 | 0.36 | 0.39 |
| Trout-perch | *Percopsis omiscomaycus* | O | 1.57 | 0.37 | 0.34 |
| Suckermouth Minnow | *Phenacobius mirabilis* | O | 1.40 | 0.38 | 0.35 |
| Bluntnose Minnow | *Pimephales notatus* | I | 0.59 | 0.34 | 0.45 |
| Fathead Minnow | *Pimephales promelas* | I | 0.59 | 0.34 | 0.45 |
| Bullhead Minnow | *Pimephales vigilax* | I | 0.59 | 0.34 | 0.45 |
| Paddlefish | *Polyodon spathula* | P | 0.30 | 1.05 | 0.49 |
| White Crappie | *Pomoxis annularis* | I | 0.46 | 0.34 | 0.55 |
| Black Crappie | *Pomoxis nigromaculatus* | I | 0.46 | 0.34 | 0.56 |
| Blue Catfish | *Pylodictis olivarus* | E | 0.37 | 0.32 | 0.77 |
| Western Blacknose Dace | *Rhinichthys obtusus* | I | 0.59 | 0.35 | 0.46 |
| Sauger | *Sander canadensis* | O | 0.81 | 0.43 | 0.37 |
| Walleye | *Sander vitreus* | O | 0.67 | 0.50 | 0.39 |
| Creek Chub | *Semotilus atromaculatus* | I | 0.59 | 0.35 | 0.46 |
|  |  |  |  |  |  |

Taxa not identified to species were hybrid striper, Ictiobinae, *Ictiobus* sp., *Lampetra* sp., *Lepomis* sp., *Morone* sp., *Moxostoma* sp., *Percina* sp., Petromyzontidae, *Pomoxis* sp., and shorthead-smallmouth redhorse.
